# Development, Implementation, and Usability Evaluation of the CANMI App to Monitor the Quality of Maternal and Child Nutrition Care in Primary Health Units: Mixed Methods Pilot Study

**DOI:** 10.2196/77539

**Published:** 2025-10-20

**Authors:** Soraya Burrola-Méndez, Elizabeth Hoyos-Loya, J Emilio Quiroz-Ibarra, Cecilia Perez Navarro, Isabel Omaña-Guzmán, Jorge Ángel González-Ordiano, Omar Acosta-Ruiz, Arturo C Bautista-Morales, Daniela Tánchez Sandoval, Sonia Hernández-Cordero, Cinthya Muñoz-Manrique, Monica Ancira-Moreno

**Affiliations:** 1 Health Department, Iberoamerican University, Prolongación Paseo de la Reforma 880, Lomas de Santa Fe, Mexico City, 01219, Mexico, 55 5950 4000 ext. 4649; 2 Observatorio Materno Infantil, Iberoamerican University, Mexico City, Mexico; 3 Center for Health Systems Research, National Institute of Public Health, Cuernavaca, Mexico; 4 Institute of Applied Research and Technology, Iberoamerican University, Mexico City, Mexico; 5 Pediatric Obesity Clinic and Wellness Unit, Hospital General de México "Dr. Eduardo Liceaga", Mexico City, Mexico; 6 Center for Evaluation and Surveys Research, National Institute of Public Health, Cuernavaca, Mexico; 7 Research Center for Equitable Development (EQUIDE), Iberoamerican University, Mexico City, Mexico; 8 Nutrition and Bioprogramming Coordination, National Institute of Perinatology, Mexico City, Mexico

**Keywords:** mobile health technologies, mHealth technologies, maternal and child nutrition, quality care, mobile app, primary health care

## Abstract

**Background:**

In Mexico, the maternal and child population continues to face a high burden of malnutrition, posing a persistent public health challenge. The health care system plays a crucial role, not only in addressing existing cases but also in preventing and detecting malnutrition early. Mobile health technologies have the potential to strengthen maternal and child health services by improving the quality, accessibility, and timeliness of nutritional care.

**Objective:**

The aim was to design, develop, and assess the usability and acceptance of a mobile app**—**CANMI (Calidad de la Atención Nutricional Materno Infantil; its Spanish acronym)—to monitor the quality of maternal and child nutritional care in primary health care units in Mexico.

**Methods:**

The framework of the CANMI app was based on 16 validated indicators designed to assess the quality of nutritional care during the preconception, pregnancy, postpartum, early childhood, and preschool stages. The app was developed for both iOS and Android systems using a user-centered design approach. Following development, we conducted a pilot usability study in a randomized sample of 18 primary health care units in Guanajuato, Mexico. Trained nutritionists implemented the app and collected usability data at the end of the initial use period and again 6 weeks later. To further explore user experience, semistructured online interviews were conducted to identify barriers, facilitators, and overall satisfaction with the app.

**Results:**

The CANMI app allows the systematic registration of key indicators to assess the quality of nutritional care in primary health care settings. Users described the app as simple, intuitive, and visually appealing. Overall usability was rated positively, with a mean score of 71.13 (SD 11.68) on the System Usability Scale, indicating good acceptability. The app’s offline functionality, streamlined interface, and efficiency in data collection were identified as key facilitators of use. Reported benefits included reduced time for data entry and perceived improvements in the quality of nutritional care. Identified barriers to integration included the need to use personal devices, user fatigue due to prolonged screen time, inconsistent clinical records, and limited time to incorporate the app into routine workflows. Importantly, the app encouraged and promoted improvements in documentation practices and heightened awareness among health personnel regarding the precision and clarity of their nutritional recommendations.

**Conclusions:**

The CANMI app provides a feasible and effective solution for monitoring the quality of maternal and child nutritional care in primary health settings. Its high usability and offline capabilities make it particularly suitable for low-connectivity environments. Beyond facilitating data collection, the app contributed to improved clinical documentation practices and enhanced health care provider awareness of care quality. Consequently, the app represents a promising digital tool to support the implementation of evidence-based, user-centered strategies aimed at strengthening maternal and child health services in resource-limited contexts.

## Introduction

### Background

Proper nutrition during the perinatal period and early childhood is critical to preventing malnutrition and its long-term impacts on mothers and children [1]. In Mexico, malnutrition remains a major concern: among children aged less than 5 years, rates of underweight, stunting, wasting, and obesity are significant [2], while many women of reproductive age face anemia, overweight, and obesity [3,4]. Most of these issues can be addressed through primary care, where outpatient services play a key role in health promotion, prevention, and treatment, especially during the antenatal and perinatal periods, which are largely managed outside hospital settings [5].

High-quality health care involves accurately identifying health needs and implementing comprehensive strategies to address them through appropriate resource allocation [6]. Quality is a measurable attribute of health systems that is assessed through key dimensions such as person centeredness, effectiveness, efficiency, equity, comprehensiveness, timeliness, safety, and sustainability and must be ensured across all levels of care, from prevention to specialized services [7,8].

In Mexico, efforts to define and monitor the quality of nutritional care have led to the development of 16 indicators focused on maternal and child health at the primary care level, which address up to 85% of the health needs of the mother-child dyad [9,10]. However, 2021 data from 6 states showed that only 8 out of 100 women and children aged less than 5 years received quality nutritional care in primary care settings. Major barriers identified by health care providers included insufficient training in malnutrition prevention, diagnosis, and treatment, especially among medical and nursing staff, and the lack of a system to monitor the quality of nutritional care [11,12].

Efforts to improve the quality of health care, particularly in maternal and child nutrition, face challenges such as insufficient training and the lack of monitoring systems [11,12]. Mobile health (mHealth) offers clear advantages over conventional monitoring systems by enabling continuous, real-time health monitoring and improving accessibility, especially for remote populations. It reduces long-term costs by minimizing the need for physical infrastructure and manual labor and enhances health care delivery by enabling real-time monitoring, identifying care gaps, and providing accessible, personalized information [13,14]. These technologies promote care coordination, support clinical decision-making, and improve communication between health care professionals, ultimately leading to more timely and individualized care [15,16].

This is feasible considering that 83.1% of the population has internet access, and among them, 97.2% access it via a smartphone [17]. Although it faces challenges such as technology adoption and data security, mHealth is more adaptable and better suited for integration with modern health care IT systems compared to the slower, less-flexible conventional methods. In maternal and child health, mHealth tools allow for continuous monitoring, ensuring health care providers can respond quickly and effectively to safeguard the well-being of the mother-child dyad [18].

### Objectives

Promoting research in this area is critical to unlocking the potential of mHealth technologies in optimizing nutritional care within Mexico’s public health system. This study aims to design, develop, and assess the usability and acceptance of a mobile app that will equip health care personnel with the tools to monitor and enhance the quality of maternal and child nutritional care at the primary health care level, ultimately improving health outcomes for the mother-child dyad.

## Methods

### Design and Development of the App

The design and development of the CANMI (Calidad de la Atención Nutricional Materno Infantil) app were based on 2 main components. The first includes the needs, strengths, and gaps identified through a previous diagnostic evaluation of the quality of maternal and child nutritional care in Mexico [11]. The second, a group of 16 validated quality indicators, organized across 5 stages of the life course**—**preconception, pregnancy, postpartum, infancy, and preschool age**—**specifically developed for this context by Ancira-Moreno et al [9].

The development phase involved defining the app’s conceptual framework, designing user interfaces and screens, and iteratively refining the prototype. The app was programmed for both iOS and Android operating systems. Quality levels within the app were categorized based on compliance with the indicators: ≥90% indicates good quality nutritional care, 71% to 89% indicates deficient nutritional care, and ≤70% indicates poor quality nutritional care. A traffic light system was used to visualize the results.

**Figure 1.**
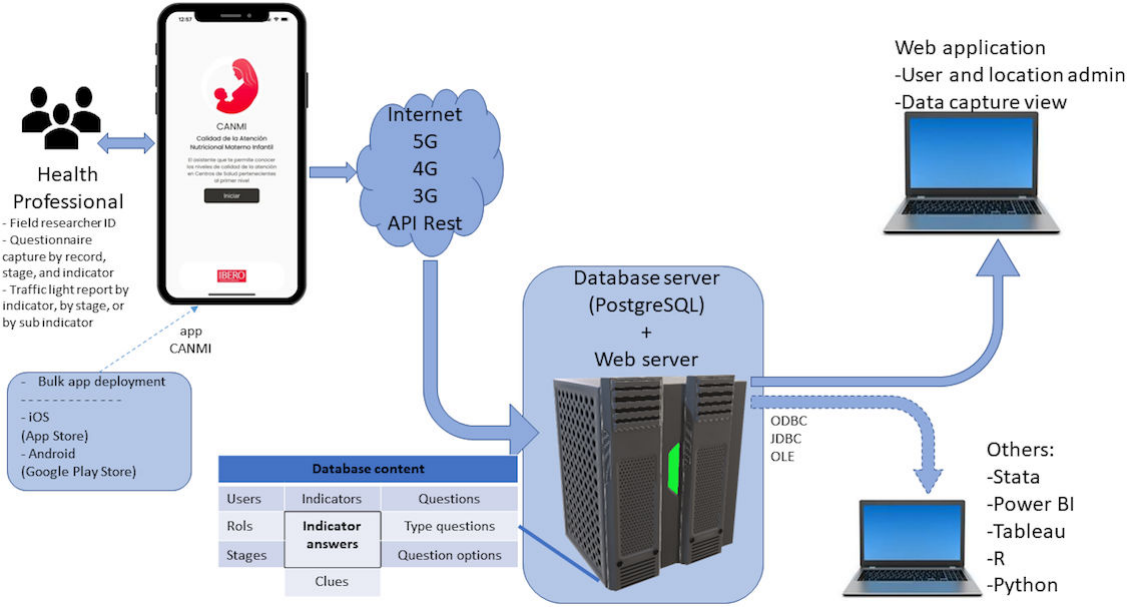
App workflow. Process of collecting, storing, and transmitting quality-indicator data in the mobile app. API: application programming interface.

Figure 1 illustrates the app’s workflow. The front end allows health professionals or quality care monitors to register responses to a stage-specific questionnaire based on the quality indicators. Once the data are captured, they are stored locally on the device. When a minimum of 3G internet connectivity becomes available, the data are automatically transmitted to a centralized server using a representational state transfer application programming interface, which enables secure and efficient bidirectional communication between the device and the server.

On the server side, a centralized database stores all responses and generates quality indicators linked to the user, health center, life stage, and question type. The mobile app allows users to intuitively visualize these results and corresponding quality metrics. Furthermore, a web-based app is connected to the server to facilitate data review, ensuring accuracy and completeness. The server architecture also supports integration with external analytics and reporting tools—such as Stata (StataCorp LLC), Tableau (Tableau Software LLC), and R (R Foundation for Statistical Computing)—via Open Database Connectivity, Java Database Connectivity, or an Object Linking and Embedding interface, allowing for flexible data analysis and reporting.

### Usability and Acceptance Evaluation

#### Study Design and Setting

This pilot usability study was conducted in July 2024 across 18 primary health care units in Health Jurisdiction 1 of Guanajuato, Mexico—a geographic division that groups regions to organize and coordinate health care units and services—covering both rural and urban settings. A proportional allocation strategy was used to ensure the representation from each setting, and simple random sampling was applied within each stratum.

#### Participants

Eleven dietitians assigned to the Health Jurisdiction 1 of Guanajuato were invited to participate by email. Inclusion criteria required being a health professional with formal training in nutrition and providing direct care to the population in primary health care services within this jurisdiction. Nine dietitians met these criteria and agreed to participate.

#### Implementation

Prior to the implementation, all participants provided written informed consent and subsequently completed a training program (T0: preimplementation assessment) conducted over 2 consecutive days. The training aimed to ensure consistent use of the CANMI app and to improve the accuracy of data collection.

During the first session, the app was installed on the participants’ personal mobile devices, and an overview of its key functionalities, navigation structure, and the content of the 16 validated quality indicators was provided. In the second session, participants engaged in guided practice using real-world data extracted from existing patient records. A total of 150 records were reviewed, comprising 30 records from each life stage. All exercises were performed in offline mode, with subsequent synchronization of data once a network connection became available. To assist participants, a detailed user guide was distributed, which included instructions on app installation, indicator navigation, and data submission procedures.

Software refinements were implemented iteratively based on feedback received during the training phase. Following the training, participants implemented the app in one of the 18 assigned health care units to assess the quality of maternal and child nutritional care. This implementation took place during the 6 weeks after the training period.

### Data Collection and Analysis

A mixed methods approach was used to evaluate the usability, efficiency, and user satisfaction associated with the CANMI app among participants. Usability was quantitatively assessed using the System Usability Scale (SUS) validated by Sevilla-González et al [19]. The SUS consists of a 10-item questionnaire, where each item is rated on a 5-point Likert scale ranging from 1 (“strongly disagree”) to 5 (“strongly agree”).

After the training, all participants received a link to complete the SUS questionnaire via Google Forms. SUS scores were calculated on a scale from 0 to 100, where higher scores indicate greater usability. A CI was also estimated [20]. Statistical analysis was performed using Stata (version 14.2).

Qualitative data were collected through 14 semistructured interviews with the participants. The interview guides were designed from a phenomenological approach to explore their experiences and perceptions regarding app usability [21]. To capture changes throughout the implementation process, 2 different interview guides were used. The T0 guide included contextual questions related to the participants’ workplaces, their professional experience, satisfaction with the training, and expectations regarding the use of the CANMI app. The T1 (postimplementation assessment) guide focused on the participants’ actual user experiences, perceptions of the app’s functionality, and the factors influencing the app’s adoption in their daily practice. This sequential design facilitated a structured comparison between anticipated and actual experiences, thereby enhancing the depth and robustness of the qualitative findings.

All 9 participants were invited to participate voluntarily via email at both time points. One participant did not respond at either time point (T0 and T1), and 3 others did not respond at T1. Therefore, 8 interviews were conducted at T0, and 5 were conducted at T1, with 5 nutrition personnel participating at both time points. All interviews were conducted remotely, via an online communication platform (n=12) and 2 by telephone. All interviews were conducted in Spanish, the primary language of the participants. Translations into English were performed by the research team, and accuracy was verified through a back-translation process reviewed by bilingual experts. The average duration of the interviews was 32 (SD 5) minutes.

With informed consent, interviews were audio-recorded to ensure information fidelity. Transcripts were coded using a deductive framework based on predefined categories, where the information was extracted directly from the audio recordings. To ensure confidentiality, all transcripts were anonymized by removing any personal identifiers. A thematic analysis [22] was conducted, addressing questions regarding app usability, facilitators of use, barriers to use, and improvements in the quality of nutritional care processes resulting from the app’s use.

We used the SQUIRE (Standards for Quality Improvement Reporting Excellence) reporting guideline to draft this manuscript and the SQUIRE reporting checklist when editing, which are included in Multimedia Appendix 1 [23,24].

### Ethical Considerations

This study was reviewed and approved by the Research Ethics Committee of the Universidad Iberoamericana in Mexico City (172/2022). Additionally, all procedures related to the development, training, and evaluation of the app implementation adhered to the procedures established by the authorities of the Ministry of Health of the state of Guanajuato.

Prior to participating in the training activities and interviews, all participants provided a written informed consent, which clearly stated the objectives of the study, the procedures involved, the intended use of the data, and how confidentiality would be protected. All data collected were deidentified to ensure participant privacy and confidentiality. No personal identifiers were included in the dataset. Participants did not receive any form of compensation for their participation.

## Results

### App Development Outcomes

The CANMI app was developed as a mHealth tool to support the monitoring and evaluation of the maternal and child nutritional care quality within primary health care units across the 32 federal entities of Mexico. This app provides real-time assessment service quality across 5 key life stages—preconception, pregnancy, postpartum, infancy, and preschool age—based on 16 validated indicators.

**Figure 2.**
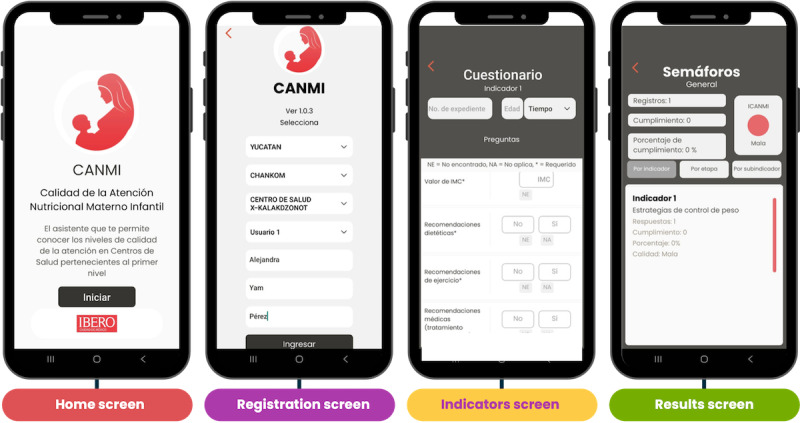
Output screens from the CANMI (Calidad de la Atención Nutricional Materno Infantil) app.

To facilitate interpretation by users, the app uses a color-coded “traffic light” system: green indicates high-quality care (≥90% compliance), yellow indicates deficient quality (71%‐89%), and red denotes poor quality (≤70%). The CANMI app was programmed to operate on both iOS and Android platforms and is designed to function offline, ensuring utility in areas with limited or no internet connectivity. Selected screenshots from the app’s user interface, which highlight its intuitive design and visual feedback mechanisms, are presented in Figure 2.

### Usability of the CANMI App

Overall, user satisfaction with the app was positive. Participants described the app as intuitive, user friendly, and visually appealing, highlighting the clarity of its screens and the logical navigation structure. During interviews, participants emphasized that the icons representing each life stage helped them quickly identify relevant indicators, which facilitated efficient data entry.

However, during the initial implementation phase, some users experienced confusion when selecting indicators. This confusion primarily stemmed from their unfamiliarity with the structure and content of the 16 indicators designed to assess the quality of maternal and child nutritional care at the primary care units. As a result, there were occasional misclassifications of data under incorrect indicators. To address this issue, a concise guide outlining all indicators and subindicators by life stage was developed and distributed. This significantly improved both the accuracy and ease of use in subsequent data capture at the centers.

Participants reported that the app’s interface and core functionalities were appropriate and did not require substantial adjustments. However, some suggestions for improvement were offered, including (1) limiting data entry access to only the relevant life stage indicators based on the user’s patient population to reduce errors and (2) enabling batch submission of multiple records, rather than requiring each entry to be uploaded individually.

Regarding data submission, most users were able to use this function without complications. Only 2 (18%) participants did not use this function due to unreliable internet access at their assigned health centers. In contrast, 1 (9%) participant mentioned that internet connectivity was made available at personal expense by health care personnel. A single technical issue was reported when a user attempted to collect data across 2 facilities; upon uploading both records, the first was overwritten. This issue was reported and subsequently resolved through an app update.

However, participants noted that the current process of uploading records one by one—particularly in cases with high volumes (eg, 90 entries)—was time-consuming, although each submission was quick and straightforward. A bulk upload feature was recommended to streamline this process.

Regarding perceived effectiveness, one of the app’s most valued attributes was its capacity to generate immediate results, contributing to time efficiency in quality assessment. However, some participants were unaware of this feature due to gaps in training and instead expected the jurisdiction to provide the results.

These qualitative findings were consistent with quantitative usability results. The mean SUS score was 71.13 (SD 11.68) points (95% CI 62.42‐81.95), classifying the app as acceptable. Notably, female participants reported slightly higher usability scores (mean 72.18, SD 9.87) compared to male participants (mean 68.33, SD 12.11; Table 1).

**Table 1.** Total score and scores by sex on the usability scale of the CANMI (Calidad de la Atención Nutricional Materno Infantil) app (N=11).

	Participants, n (%)	Points, mean (SD)	Score, 95% CI
Total	11 (100)	71.13 (11.68)	62.42-81.95
By sex			
Male	3 (27)	68.33 (12.11)	55.40-81.26
Female	8 (73)	72.18 (9.87)	62.42-81.95

### Facilitators of the Use

The main facilitator identified by participants was the app’s ability to function without an internet connection during data collection. This feature is especially valuable given the frequent lack of internet access in health centers, surrounding communities, and even some municipalities. By eliminating the need for personal data plans, this functionality helps prevent user discouragement and promotes sustained use of the app:

The great advantage is that it can be used without the internet, it’s very good because there are places where we don’t have signal.

[Dietitian, Health Jurisdiction, Guanajuato]

It’s easy to use the application because you don’t need the internet to collect the data, only when uploading the information.

[Dietitian, Guanajuato]

One of the key advantages identified by participants was the app’s potential to improve time efficiency during routine supervisory activities. In the context of Guanajuato, dietitian professionals conduct quarterly supervision of physicians and nurses, assessing quality indicators by the state health system, which can take up to a full day in each unit visited. Participants noted that integrating the CANMI app into these processes—either as the primary data collection tool or as a complementary resource—could streamline workflows by reducing the time required for manual data entry and enabling immediate and simplified transmission of results:

There’s a lot of workloads, many programs, and many indicators. If we had this app in Guanajuato to measure our indicators by unit and at the municipal level, it would be great because you just record the data, and it automatically calculates the indicator.

[Dietitian, Guanajuato]

Another reported benefit of the app was that it can help make immediate improvements in nutritional care. Participants noted that the training itself, combined with continuous use of the tool, encouraged self-reflection and greater awareness of care gaps, particularly regarding missed counseling opportunities and incomplete documentation in medical records. One of the dietitians emphasized that the app could also serve as a self-assessment tool for physicians and nurses, fostering behavior change and improved practices across the broader care team:

I do see the app (CANMI app) as something that can help us improve the quality of care, but I feel that all staff should be involved because only the dietitians were given the app, and we are not in all the UMAPS (Primary Health Care Medical Units), I can’t go to the field every day, it’s impossible. Doctors and nurses should also use it because they need to evaluate themselves, ‘hey, I’m not recording how much dose,’ now it’s like ‘here are the files, and you do it.’ It’s important to involve them to keep improving.

[Dietitian, Guanajuato]

### Barriers to Use

Several barriers were identified that may complicate the optimal use of the app. A key concern was the reliance on personal mobile devices for quality assessment. While most participants reported no major issues using their phones, some expressed discomfort and noted that this could discourage broader adoption among other health professionals. The lack of institutional devices was seen as a limitation, particularly because using the app temporarily disables access to messages and notifications, generating anxiety for some users.

Additionally, the prolonged use of the app—averaging 60 minutes per session—was described as physically tiring, especially when completing multiple entries consecutively:

It’s very tiring to do it on the cell phone, I mean, it’s fine, but after a while, it gets tiring because it’s small, it would be better on a tablet or a computer ... The ideal would be for the health center to provide the equipment, because I have e-books (on my phone) and in training, they suggested deleting some (so the app would work better), and I said “Oh, no, it doesn’t seem right to me, it’s a book I can’t erase,” but if they gave us exclusive equipment for data entry, it would be fabulous.

[Dietitian, Guanajuato]

Another barrier to app use was the mismatch between the specificity of the app’s indicators and the way health personnel typically document patient records. All interviewees mentioned that clinical records often lack detailed information, such as recommended dosages or specific actions. A frequently cited example was the indicator for physical activity counseling in preschool-aged children, which requires documentation of both the type and duration of activity. In practice, staff often record generic phrases such as “recommended exercise” or “recommended physical activity.” As a result, half of the interviewees who referenced this example marked the indicator as completed in the app, while the other half did not. These inconsistencies were often noted in the app’s observation field:

I had no hesitation in marking the fulfillment of the very specific indicators. For example, if the medical note had a general recommendation, I put in the app that it didn’t meet the requirement, because “recommended healthy eating” is not adequate guidance. Then I would note in the observations that it was general.

[Dietitian, Guanajuato]

I found general notes like “recommended correct eating” or “continue with the same eating plan” or “promote physical activity,” that’s why in the app sometimes we ended up with files where everything was marked as “no.” But sometimes it happens that it’s not that health staff don’t give complete or accurate recommendations, but we have formats that are closed and don’t allow that wording

[Dietitian, Guanajuato]

A similar issue was found with postpartum indicators, such as breastfeeding guidance, which requires specifying recommendations for latch or breast massage. However, clinical notes from medical, nursing, and even nutrition staff typically document only general statements such as “breastfeeding guidance provided,” limiting accurate reporting:

The app asks very specifically, and we only put “breastfeeding recommendation,” but it’s not specified point by point what we told them because it would take us more time to fill out the note, and since we don’t always have the printer at the health center or there’s no ink, we have to do it by hand, and that takes more time.

[Dietitian, Guanajuato]

Limited time available was identified as a key challenge for using the app. Although entering indicators took only an average of 5 to 10 minutes per record, depending on the life stage, coherence, or legibility of notes, completing the target of 30 records per stage, along with transportation and files retrieval for some users, required 1 or 2 full days of work. This pace of use significantly affected their patient agenda. This workload can disrupt clinical schedules, representing a barrier to integrating app use into daily routines.

### Improvements in the Maternal and Child Nutritional Care Through CANMI App Use

The use of the app has led to improvements in maternal and child nutritional care, as reported by dietitians. Some participants noted that the app facilitated more precise and clear recommendations, improving user understanding and the accuracy of documentation. For example, instead of simply recording “supplementation recommended,” they now document “recommended folic acid at this dosage,” or instead of “breastfeeding counseling provided,” they specify “guidance on latch provided.”

**Figure 3.**
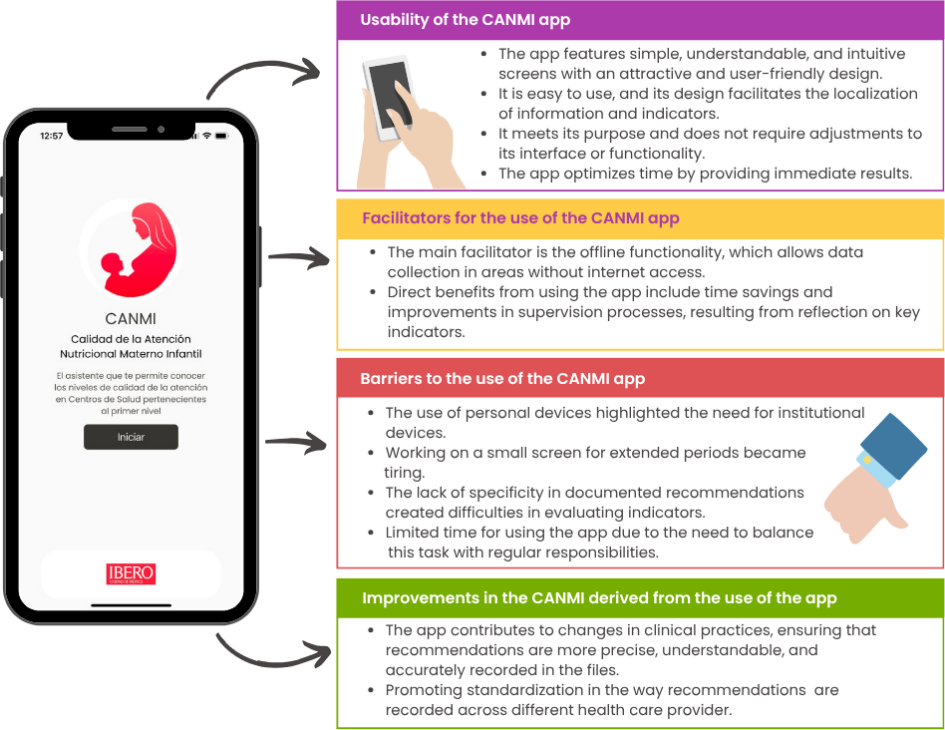
Key elements identified in each category analyzed about the CANMI (Calidad de la Atención Nutricional Materno Infantil) app.

In addition, efforts to standardize documentation practices were initiated among nursing staff. One participant proactively engaged with 2 of the 3 nurses working at a health center, providing guidance on how to fill out notes more precisely and standardize records. This initiative was framed as a means to improve the quality of care. Participants expressed the expectation that these improvements would ultimately translate into better health outcomes for women and children. Figure 3 summarizes the main elements identified in each of the analyzed categories: usability, facilitating factors, barriers to the use, and improvements.

## Discussion

### Principal Findings

The CANMI app is a tool developed in response to the specific needs of the Mexican health care system, designed to monitor and evaluate the quality of nutritional care across 5 life stages: preconception, pregnancy, postpartum, infancy, and preschool years. Its design considers the availability of local resources within health care units for effective use, such as the lack of internet access in health care facilities, enabling data collection in areas without access to this service. This lack of internet access has been identified as one of the main factors hindering the implementation of information technologies in the field of mHealth, particularly in areas that lack internet access or have weak signals [25].

One key finding from the implementation of the CANMI app is that its interface is simple, understandable, and intuitive, with an attractive and user-friendly design. This is crucial for its successful adoption by health care professionals. The ease of use and accessibility of the interface are widely recognized as determining factors for the effectiveness of health technologies, especially in environments with diverse personnel and varying technological capabilities. This is supported by a systematic review by Jakob et al [26] who emphasized the importance of a user-friendly and technically stable app design as a positive factor for adherence to mHealth apps.

Regarding the effectiveness of the app in meeting its objective, it is important to note that feedback received indicates that no adjustments are necessary for its interface or functionality. This stability is essential to building trust among users, as frequent changes or functional issues can discourage the use of the tool [27,28]. Moreover, functional stability not only enhances the user experience but also facilitates the ongoing evaluation of the tool, as the data collected are more consistent and reliable.

Visual fatigue due to the use of small screens is a specific problem that adds to broader concerns about the usability of the CANMI app and was identified as a key barrier to the routine adoption of the CANMI app by health care professionals. Additionally, a lack of standardization in documenting recommendations and instructions within clinical records was identified. While this does not cause significant difficulties in recording the variables corresponding to each indicator, this lack of alignment between the information recorded in medical notes and the app’s indicators can lead to inconsistencies in evaluation results. This finding aligns with what was noted by Juárez et al [29], who documented that standardization of information in clinical records in Mexico faces considerable challenges, particularly in terms of interoperability and data quality. In this context, it is crucial to implement strategies that improve the quality of medical records, with continuous training for health care personnel being a key tool in achieving this improvement [30].

Following the analysis of usability and acceptance of the app in the health care units, it was identified that the tool can contribute to generating positive changes in how health care professionals provide nutritional care services. They reported being more rigorous and specific in their records and recommendations. This finding aligns with what Saez et al [18] documented, highlighting that monitoring data on maternal and child populations helps health care workers supervise and manage care more effectively, enabling them to make key decisions to improve the health of both mother and child. Similarly, in Ethiopia, the implementation of mobile solutions in the primary health care system has improved the timely identification and registration of pregnant women and adherence to treatment protocols [31]. However, it is important to emphasize that changes in care and nutritional status do not occur automatically and require ongoing intervention and detailed follow-up.

### Limitations

This study has several limitations that should be considered when interpreting the results. First, the participant sample was small and limited to health care units in specific regions, which restricts the generalizability of the findings at the state level. Furthermore, due to the study design, the long-term impact of the CANMI app on nutritional indicators could not be accurately measured, limiting the ability to assess its sustainable effectiveness. Finally, evaluations should ideally be conducted by external personnel, but the validation process relied on health care professionals from the same units.

Future steps should focus on enhancing the app’s functionality, prioritizing adjustments that improve its efficiency and usability within the context of primary health care in Mexico. Besides the contribution in monitoring maternal and child care, it is essential to implement systematic training for all health care personnel (physicians, nurses, and dietitians), leading to the improvement of health outcomes for the mother-child dyad.

### Conclusions

The CANMI app has the potential to be a valuable tool in improving maternal and child nutritional care, especially in contexts where internet connectivity is limited. Although there are numerous facilitators of use, identified barriers such as the lack of specificity in medical records, the use of personal devices, and additional workload must be addressed to ensure its long-term effectiveness and sustainability. Overall, the app has shown that it can have a positive impact on improving record-keeping practices and raising staff awareness, contributing to more accurate and efficient care. However, it is essential to continue monitoring its use and adjust based on the needs and experiences of health care personnel to maximize its potential.

## Supplementary material

10.2196/77539 Multimedia Appendix 1 Interview guide.

## Abbreviations

CANMI: Calidad de la Atención Nutricional Materno Infantil
mHealth: mobile health
SQUIRE: Standards for Quality Improvement Reporting Excellence
SUS: System Usability Scale
