# Supplementary material for: Development, Implementation, and Usability Evaluation of the CANMI App to Monitor the Quality of Maternal and Child Nutrition Care in Primary Health Units: Mixed Methods Pilot Study
Source: JMIR Form Res. 2025 Oct 20;9:e77539. doi: 10.2196/77539 (PMC12536923; doi:10.2196/77539)
Supplement: Multimedia Appendix 1 [file formative-v9-e77539-s001.docx]

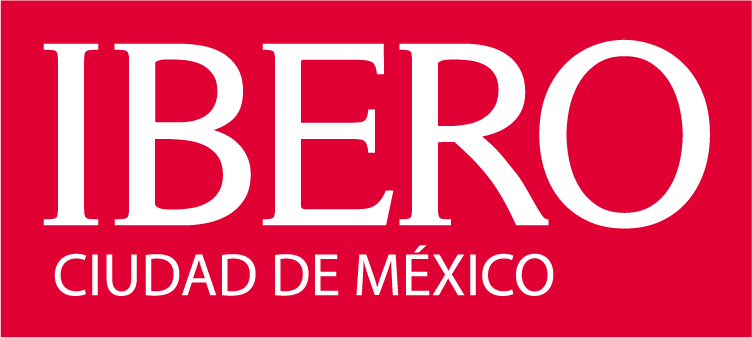


**UNIVERSIDAD IBEROAMERICANA**

**Departamento de Salud**

***Desarrollo de una aplicación para mejorar la evaluación y el monitoreo***

***de la calidad de la atención nutricional materno infantil (CANMI)***

**INTERVIEW GUIDE**

**Health Personnel**

| **Objetivo** |
| --- |
| To evaluate the use of the CANMI APP, considering indicators of acceptance, adoption, and loyalty at two points in time over a six-month period, among healthcare personnel participating in the pilot program to be conducted in healthcare centers in Guanajuato. |

| **Instructions** |
| --- |
| If the interview is conducted in person, briefly explain the objectives to the interviewee and provide them with the informed consent form. If the interview is conducted remotely, the interviewee must have received the informed consent form by email in advance and signed it by email before beginning the interview.  When starting the recording, the date, interview format, and the interviewee's position must be identified; DO NOT provide their name. Also, ask the interviewee if they agree to the interview and have it audio-recorded as a backup in case of any difficulties.  The Guide is not exhaustive, and questions may be omitted or others added if, during the interview, relevant and specific topics arise that warrant further investigation. |

| **INTERVIEW - T0** |
| --- |
| **General questions**   1. How long have you worked at this health center? 2. What is the profile of the clients you typically serve? 3. In your experience, what factors influence the quality of nutritional care? 4. How would you rate the quality of nutritional care at this health center? 5. In your opinion, what are the main challenges in providing quality nutritional care?   **Questions about training**   1. What is your overall opinion of the training received? 2. Was the objective of the CANMI App clear to you? 3. Do you have questions about what was explained about how to use the CANMI App?    1. FOR THOSE DOING THE PRACTICE: How useful was the practice session for using the CANMI App?   **Questions about App-CANMI**   1. Some people believe that technology offers tools to promote good health practices. What do you think about this? 2. Based on what you learned during the training, what benefits do you think using the CANMI App will have in improving the quality of nutritional care at this health center? 3. What do you think might be barriers to the proper use of the CANMI App?    1. Personal barriers    2. Barriers at the health center    3. Barriers within the health system 4. Do you think using the CANMI App will make your work easier or more complex? |

| **QUESTIONS - T1** |
| --- |
| **Questions about the App's Usability**   1. Did you download the CANMI App on your personal cell phone? Was it easy or difficult? 2. Do you know or use other apps to do your work? 3. Regarding the ones you mentioned (or others you know in general), how user-friendly do you find the app? 4. Do you think using the CANMI App has made your work easier or more complex? 5. How easy or difficult has it been to use it?    1. How often have you been able to use it at the health center?    2. What difficulties have you encountered using it? (If applicable)    3. Have you had any questions while using it?    4. Has anyone helped you resolve any questions?   **Questions about the operation of the App**   1. Have you collected the information offline?   ANSWER YES:   - 1. Do you know if the data was downloaded correctly when connected?   2. Did you find this feature useful?   ANSWER NO:   - 1. Why haven't you used this feature?   2. Has not being able to use it discouraged you from continuing to use it?  1. What barriers have you encountered when using the CANMI App?    1. Personal    2. Contextual 2. Have you been able to review the results the CANMI App provides on the quality of nutritional care at your health center?    1. What do you think about these results?   **Questions about App Adoption**   1. Will you continue to use the CANMI App as part of your routine activities at the health center?    1. YES: What are the reasons why you will continue to use it?    2. NO: What are the reasons why you will continue to use it? 2. What would need to happen for you to include the app in your routine activities at the health center? 3. How easily do you think it would be for other colleagues to use the CANMI App as part of their routine activities at the health center? 4. Finally, have the results that the CANMI App has yielded regarding the quality of nutritional care at your health center led you to change anything in your general and nutritional care routine for users, specifically women in the preconception, pregnancy, and postpartum stages, and mothers of children under 5 years of age? 5. Do you have any suggestions for improvements to the CANMI App? |
